# Exploring standard and low luminance visual acuity and the Moorfields Acuity Chart as outcome measures in inherited retinal disease

**DOI:** 10.1111/opo.13504

**Published:** 2025-06-02

**Authors:** Laura J. Taylor, Amandeep S. Josan, Robert E. MacLaren

**Affiliations:** 1 Nuffield Laboratory of Ophthalmology, Department of Clinical Neurosciences, University of Oxford, Oxford, UK; 2 Oxford Eye Hospital, Oxford University Hospitals NHS Foundation Trust, Oxford, UK

**Keywords:** low luminance visual acuity, Moorfields Acuity Chart, outcome measure, visual acuity inherited retinal disease

## Abstract

**Introduction:**

Standard visual acuity (VA) is often insensitive to subtle changes in vision that result from inherited retinal disease. Low luminance VA (LLVA) has grown in popularity as an alternative acuity measure. A new test, the Moorfields Acuity Chart (MAC) has been designed as a more sensitive and repeatable test for use in patients with age-related macular degeneration. The study explores the utility and repeatability of standard VA, LLVA and the MAC in a mixed cohort of patients with inherited retinal disease.

**Methods:**

Participants were recruited as part of the visual function in retinal degeneration study (Ethics Reference 20/WM/0283). Standard VA was obtained using the Early Treatment of Diabetic Retinopathy study (ETDRS) chart placed at 4 m. LLVA was obtained using the same ETDRS chart with the addition of a 2.0-log unit neutral density filter. MAC VA was obtained using standard clinic room lighting. All participants completed repeated testing.

**Results:**

Thirty-five patient participants and 36 healthy controls, with logMAR 1.00 (6/60) or better, completed testing. Both LLVA and MAC VA were reduced compared to standard VA in patient participants and healthy controls (linear mixed model: *p* < 0.001). All three acuity tests show comparable sensitivity, specificity and repeatability. A subset of participants (patient participants *n* = 34, healthy controls *n* = 35) completed microperimetry. Post hoc analysis of microperimetry volume sensitivity correlated significantly with all of the acuity tests and showed no significant difference in the gradient of the slopes. This suggests that VA, LLVA and MAC VA decline at a consistent rate with disease progression.

**Conclusion:**

All three acuity tests could be considered viable outcome measures for clinical trials. For patients with early to moderate inherited retinal disease (logMAR 1.00 (6/60) or better), no single acuity chart appeared significantly beneficial.

**Supplementary Information:**

The online version of this article (doi:10.1111/opo.13504) contains supplementary material, which is available to authorized users.

## Key points

- All three acuity tests (standard visual acuity, low luminance visual acuity and the Moorfields Acuity Chart) showed reduced visual acuity in retinal disease patients compared to healthy controls.
- All three acuity tests showed similar sensitivity and repeatability.
- Using microperimetry central retinal sensitivity as a marker of disease progression, all three acuity tests showed a consistent reduction with reduced central sensitivity.

## INTRODUCTION

Standard visual acuity (VA) using the Early Treatment of Diabetic Retinopathy study (ETDRS) chart with a standardised procedure is now a well-established component of ophthalmic clinical trials and a marker of central visual function.^1^ The standard VA test and ETDRS chart are well designed and easy to implement, requiring relatively low-cost and simple equipment. However, in patients with retinitis pigmentosa (RP) and rod-cone degeneration (common subtypes of inherited retinal disease), central visual function is often spared until late disease stages, rendering the monitoring of standard VA ineffective at detecting early disease changes. Therefore, alternative visual function measures are required to quantify the impact of new treatments for many inherited retinal diseases.^2–4^

**FIGURE 1:**
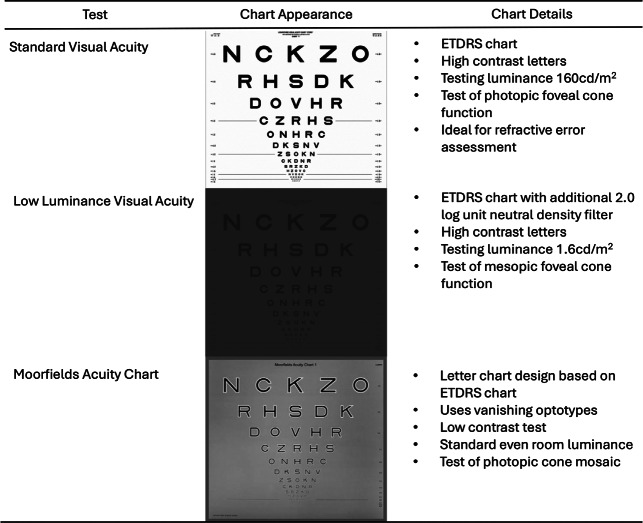
Summary of visual acuity test characteristics. ETDRS, Early Treatment of Diabetic Retinopathy study.

Low luminance VA (LLVA) was originally developed for use in age-related macular degeneration (AMD) and has grown in popularity since its first use in 1996.^5^ It uses the same equipment as standard ETDRS VA but with the addition of a 2.0 neutral density filter (precision-vision.com) (Figure 1). LLVA has been shown to enable the earlier detection of disease changes (specifically central retinal sensitivity changes) in choroideremia and retinitis pigmentosa GTPase regulator (RPGR)-associated retinitis pigmentosa (RP) than standard VA.^2^

The Moorfields acuity chart (MAC) (Figure 1) has subsequently been developed as a more sensitive and repeatable measure of early changes in central cone photoreceptor diseases such as AMD. The chart is a modified version of the ETDRS letter chart and uses vanishing optotypes, whereby once the letter is no longer recognisable it is also undetectable. This is created using a pseudo high-pass design, dark letters surrounded by lighter edges with an overall mean luminance equal to the background luminance.^6^

The ideal VA outcome measure in inherited retinal disease would be straightforward to perform, sensitive to a range of disease stages and able to detect subtle visual changes that result from disease-related degeneration or therapeutic changes. VA measures remain popular clinical trial outcome determinants and accepted regulatory endpoints for inherited retinal disease.^7^ Therefore, this study explores the utility and repeatability of standard VA, as well as alternative acuity measures (LLVA and the MAC) in a mixed cohort of participants with a range of inherited retinal diseases.

## METHODS

Healthy control and patient participants were recruited as part of the visual function in retinal degeneration study (UK research ethical approval reference: 20/WM/0283, ISRCTN24016133). A detailed study methodology is provided in the published protocol.^8^ All data were collected in accordance with the tenets of the Declaration of Helsinki at Oxford Eye Hospital, United Kingdom. The study focused on exploring outcome measures in patients with early-moderate inherited retinal disease; therefore, patients with VA worse than logMAR 1.00 (6/60) or significant co-pathologies were excluded.

All participants underwent a refraction before acuity testing by a qualified optometrist, which was worn throughout VA testing. VA tests were completed according to the standardised ETDRS VA assessment protocol.^1^ The room lights were switched off and only the retro-illuminated ETDRS chart (precision-vision.com), placed at 4 m, was switched on (luminance of 160 cd/m^2^). Participants were encouraged to read down the letter chart, left to right and down from the top of the chart as far as they could. No formal stopping rule was applied. The total number of correctly read letters was used as the VA score. A 2.0 log unit neutral density filter was added to the above set-up for LLVA testing, reducing the testing luminance to 1.6 cd/m^2^. This was chosen as it is the preferred methodology for LLVA testing.^5^ Monocular testing order, including test repeats and letter chart selection, was carefully designed to reduce bias from letter chart memorisation and optimise testing (Table S1). There was no formal dark adaptation time provided prior to LLVA testing. MAC VA was assessed last, also at 4 m, mounted onto the ETDRS chart stand but with the retro-illumination turned off and room light switched on (luminance 24 cd/m^2^).

Microperimetry was completed using the macular integrity assessment (MAIA) microperimeter (Centervue, icare-world.com) in a darkened room (light level <1.0lux) without any formal dark adaptation or pupil dilation. A 1° diameter red circle was used as the fixation target. A standard 10-2 68-point test grid was used with a 4-2 decibel (dB) bracketing threshold strategy and Goldmann size III stimulus of various intensities (0–318 candelas per metre squared (cd/m^2^)) on a mesopic background (1.27 cd/m^2^). The stimulus dynamic range was 0–36 dB. Prior to testing, subjects were informed about the test and task. The non-tested eye was occluded throughout.

### Statistical analyses

Descriptive and parametric analyses were performed using Graphpad Prism 10.3.0 (graphpad.com), including means and 95% confidence intervals (CI). VA results for each letter chart were assessed via Bland–Altman analyses using data from one eye per participant. Linear mixed model analysis was undertaken in R (r-project.org/) using the lme4 package to account for paired and nested data (including data from both eyes) to maximise the statistical power of the analyses.^9^ To compare how letter score varied with microperimetry mean sensitivity across all three chart/test types, a linear mixed model was constructed with mean sensitivity as the dependent explanatory variable, letter score and examination type as the independent predictor variables along with a letter score/examination type interaction term. Subject ID was set as the random intercept term to account for data nesting since each participant performed all three tests where possible. To compare the prediction sensitivity and specificity of each examination type and its ability to differentiate between patient and control groups, logistic regression was employed using the core ‘glm’ function with a binomial fitting function and receiver operating characteristic (ROC) curves generated using the pROC function.^10^

## RESULTS

### Demographics and participant characteristics

Seventy-one eyes from 36 healthy participants and 68 eyes from 35 patient participants, of similar age, diagnosed with an inherited retinal disease completed testing (Table 1). The patient cohort comprised mainly individuals diagnosed with choroideremia (*n* = 12), *RPGR*-associated RP (*n* = 6), Usher syndrome type II *(USH2A)*-associated RP (*n* = 7) and *Rho*-associated RP (*n* = 3), which all present typically as a rod-cone degeneration. Figure S1 shows the full range of genotypes of patient participants. All eligible eyes from patients and healthy controls completed testing. One late-disease stage patient participant could not resolve any letters with LLVA but could carry out VA and MAC VA testing; they had a VA of logMAR 1.00 (6/60).

**TABLE 1:** Participant demographics and acuity summary descriptives.

	Controls	Patients
*n*	36	35
M:F	17:19	25:10
mean age, years (SD, range)	29.4 (14.8, 19–74)	37.4 (17, 17–57)

	OD	OS	OD	OS
*n* (eyes)	36	35	34	34
VA, ETDRS letters, mean (SD)	91.0 (5.0)	92.0 (3.8)	77.3 (9.9)	75.4 (9.7)
LLVA, ETDRS letters, mean (SD)	80.3 (5.4)	80.3 (3.5)	66.1 (15.4)	64.2 (16.4)
MAC VA ETDRS letters, mean (SD)	73.0 (5.1)	74.1 (4.2)	57.2 (12.3)	55.2 (10.8)
VA-LLVA difference, ETDRS letters, mean (SD)	10.8 (4.6, 1–23)	11.3 (4.0, 0–23)	11.2 (8.5, 0–50)	10.9 (9.9, 0–50)
VA-MAC VA difference, ETDRS letters, mean (SD, range)	18.0 (3.0, 13–23)	17.4 (4.8, 0–28)	20.1 (6.2, 0–33)	19.7 (5.3, 0–28)
LLVA-MAC VA difference, ETDRS letters, mean (SD, range)	7.3 (3.6, −1.0 to 15)	6.1 (3.5, 0–15)	8.9 (8.1, −26 to 19)	8.5 (9.3, −29 to 44)

Abbreviations: CI, confidence interval of mean; ETDRS, Early Treatment of Diabetic Retinopathy screening; F, female; LLVA, low luminance VA; M, male; MAC, Moorfields Acuity Chart; OD, right eye; OS, left eye; SD, standard deviation; VA, visual acuity.

### Acuity analyses

Both LLVA and MAC VA absolute values were significantly lower than standard VA values in both patient participants and healthy controls (Satterthwaite: *p* < 0.001) (Table 1). While MAC VA demonstrated the lowest range of values of all three acuity test types, LLVA showed the greatest range of values in patient participants, followed by MAC VA. Despite this, the mean low luminance difference (VA – LLVA) and MAC difference (VA – MAC VA) values were comparable between patient participants and healthy controls in both eyes, suggesting VA values reduce alongside LLVA and MAC VA values.

**FIGURE 2:**
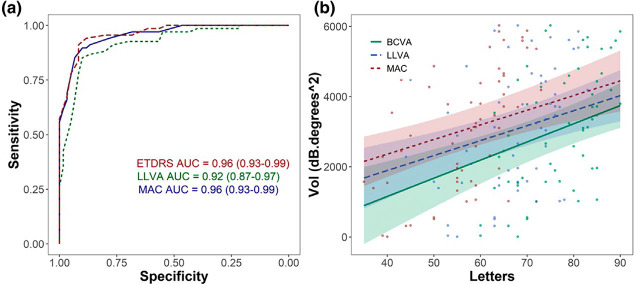
(a) Comparable receiver operating characteristic curves for Early Treatment of Diabetic Retinopathy (ETDRS) visual acuity (VA), low luminance VA (LLVA) and Moorfields Acuity Chart (MAC) VA. Area under the curve (AUC). (b) Comparable correlation analyses between microperimetry volume sensitivity and letter score for each VA test.

To investigate test sensitivity and specificity, the receiver operating characteristic (ROC) curves were plotted. All ROC plots showed high sensitivity and specificity, with comparable areas under the curve (AUC) for all three test types. Both VA and MAC VA showed comparable confidence intervals, and the LLVA AUC showed slightly wider confidence intervals, but overall, there were no statistically significant differences between all three tests (bootstrapping: *p* > 0.05) (Figure 2a).

**FIGURE 3:**
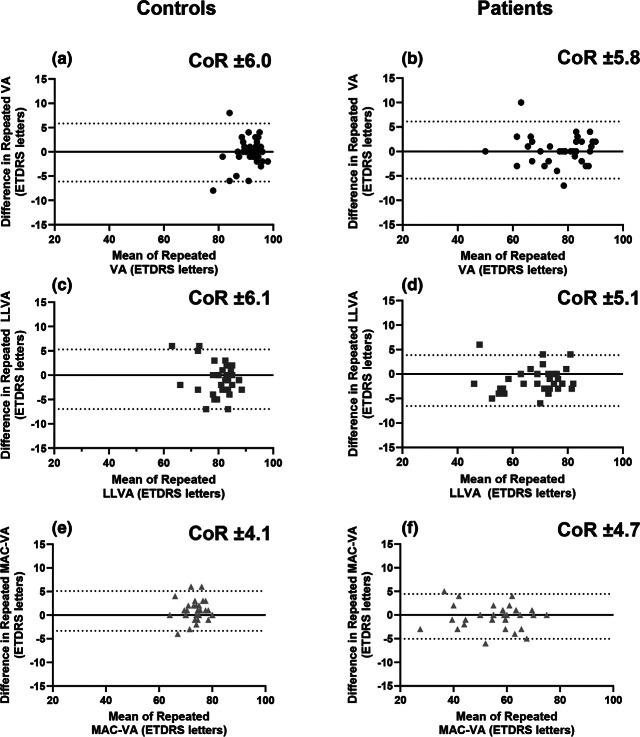
Comparable Bland–Altman repeatability plots for right eye Early Treatment of Diabetic Retinopathy (ETDRS) visual acuity (VA), right eye low luminance VA (LLVA) and left eye Moorfields Acuity Chart (MAC) VA for both healthy controls (a, c, e) and patient participants (b, d, f). CoR, coefficient of repeatability.

All participants (patients and healthy controls) completed repeated right-eye VA, right-eye LLVA and left-eye MAC VA testing (Table S2). Bland–Altman repeatability revealed MAC VA had a marginally lower coefficient of repeatability, one letter in the right eye and two letters in the left eye, compared to standard VA and LLVA (Figure 3).

### Microperimetry analyses

A subset of participants (patient participants *n* = 34, healthy controls *n* = 35) completed microperimetry. One patient participant only completed right eye testing as they were too fatigued to continue with left eye testing. Four right eye tests and three left eye tests were excluded due to >30% fixation losses (*n* = 2) or unstable fixation (*n* = 5). Two healthy control tests (one right eye and one left eye) were excluded due to >30% fixation losses. Overall, 60 patient participants and 68 healthy control microperimetry tests were included in these analyses. Mean volumetric microperimetry sensitivity for healthy controls was 6144 dB.deg^2^ (95% CI 6043–6245) and 6181 dB.deg^2^ (95% CI 6071–6294), and for patient participants 3125 dB.deg^2^ (95% CI 2471–3779) and 2940 dB.deg^2^ (95% CI 2316–3563) for right and left eyes, respectively.

VA, LLVA and MAC VA letter scores each correlated significantly with microperimetry volume sensitivity (Kenward-Roger: *p* < 0.001) (Figure 2b). Using microperimetry as a surrogate marker of disease severity, all three letter charts showed a significant decline in VA with greater disease severity (lower microperimetry sensitivity). Further analyses using linear mixed models showed that there was no significant difference between the gradients of each slope (Tukey: *p* > 0.05) suggesting VA, LLVA and MAC VA decline in a consistent manner with disease progression.

## DISCUSSION

All three VA tests showed high precision in the detection of patients with reduced visual function and comparable repeatability. More interestingly, although LLVA and MAC VA showed greater reductions in the patient group, their decline with disease progression appears consistent.

Previous studies have suggested LLVA and MAC VA to be more sensitive markers of reduced photoreceptor sampling density due to AMD.^4,5^ For intermediate AMD, ROC curves showed marginal improvement in sensitivity with LLVA = 0.83 (95% CI: 0.73–0.93) and MAC VA = 0.83 (95% CI: 0.73–0.93) compared to standard VA = 0.73 (95% CI: 0.61–0.85).^3^ However, for early-stage inherited retinal disease, this does not appear to be the case.

The reported test–retest variability for VA and LLVA compared well to those previously reported in inherited retinal disease (±7.2 letters and ± 6.7 letters, respectively) and healthy controls (±7.0 letters and ± 6.0 letters, respectively).^2^ The MAC VA test–retest variability results were similar to those previously reported, showing only modest improvements. These included ±0.10 logMAR (±5.0 letters) for healthy controls and ± 0.09 logMAR (±4.5 letters) for patients with AMD.^4^ Overall, test–retest variabilities did not appear to be affected by retinal disease. The MAC VA chart was designed to reduce variability arising from variable ending criterion, stopping rules or variation in participant ability or willingness to ‘guess’ letters at the threshold. However, given the observed improvements in test–retest variability were only small (around 1–2 letters), it suggests that the end criterion is only a minimal source of variability, when participants are encouraged to do their best and pushed to achieve their threshold acuity. Trying to ‘guess’ at the threshold and inducing forced errors to achieve a particular stopping rule is often uncomfortable for participants and the results presented here suggest it may be unnecessary.

There were several study limitations. Although the testing order was carefully designed to minimise letter chart memorisation and optimise testing procedures, the order of testing for the three acuity tests was not randomised, which could result in learning and fatigue effects within the data. However, given that participants are typically familiar with the standard VA tests and since the tests are straightforward and relatively quick to understand, any learning or fatigue effects are likely to be modest. Another limitation occurred with MAC VA repeats; this was only undertaken with left eyes, due to concerns with letter chart memorisation, whereas VA and LLVA repeats were completed with right eyes. Furthermore, only standard room lighting was used for MAC VA testing, which was dimmer than previously reported in the literature (50.5 cd/m^2^) making comparisons to previous studies difficult.^4^ This mixed inherited retinal disease patient cohort may limit the ability to identify disease-specific trends, despite most showing rod–cone degenerate phenotypes. The range of the visual acuity data is limited in the patient group since this study specifically focused on earlier stage patients (VA logMAR 1.00 (6/60) or better). Therefore, the study findings only reflect early disease-stage patients. A previous study reported a sudden drop in LLVA values when VA dropped below logMAR 0.70 (6/30, 50 ETDRS letters) in a cohort of patients with choroideremia and *RPGR*-associated RP.^2^ Only one patient participant in this study with VA of logMAR 1.00 (6/60) showed a floor effect with LLVA. Overall, there was no significant differentiation from standard VA with either LLVA or MAC VA. Further research is required to investigate the MAC VA changes in patients with more advanced disease.

## CONCLUSION

All three visual acuity tests show high sensitivity and specificity and comparable repeatability; therefore, all three could be considered viable outcome measures for clinical trials for inherited retinal disease. For early-stage inherited retinal disease patients with VA logMAR 1.00 (6/60) or better, indicating preserved central function, no single acuity chart appeared overly beneficial.

## Supplementary Information

Supplementary file (DOCX 27.2 KB)
