# Supplementary material for: Exploring standard and low luminance visual acuity and the Moorfields Acuity Chart as outcome measures in inherited retinal disease
Source: Ophthalmic Physiol Opt. 2025 Jun 2;45(5):1158–63. doi: 10.1111/opo.13504 (PMC12153033; doi:10.1111/opo.13504)
Supplement: Supplementary file 1 — Supplementary file (DOCX 27.2 KB) [file 44402_2025_4505014_MOESM1_ESM.docx]

*Table S1: Acuity test sequence and letter chart rotation.*

| Acuity testing sequence and letter chart rotation |
| --- |
| 1. Refraction of both eyes, using chart R |
| 2. Right eye LLVA, left eye occluded, chart 1 |
| 3. Right eye ETDRS VA, left eye occluded, chart 1 |
| 4. Left eye LLVA, right eye occluded, chart 2 |
| 5. Left eye ETDRS VA, right eye occluded, chart 2 |
| 6. Repeat right eye LLVA, left eye occluded, chart R |
| 7. Repeat right eye ETDRS VA, left eye occluded, chart R |
| 8. Left eye MAC VA, right eye occluded chart 2 |
| 9. Right eye MAC VA, left eye occluded, chart 1 |
| 10. Repeat left eye MAC VA, right eye occluded, chart 2 |

*Table S2: Summary descriptives for repeat acuity values.*

|  | Controls | Patients |
| --- | --- | --- |
| Repeat VA, ETDRS letters (95% CI) | 91.1  (89.7-92.6) | 77.0  (62.0-72.8) |
| Repeat LLVA, ETDRS letters (95% CI) | 81.1  (79.0-83.2) | 67.4  (62.0-72.8) |
| Repeat MAC VA, ETDRS letters (95% CI) | 73.2  (71.8-74.6) | 55.5  (57.7-59.3) |
| *Visual acuity: VA, Early Treatment Diabetic Retinopathy Study: ETDRS, Confidence interval of mean: CI, low luminance VA: LLVA, Moorfields Acuity Chart: MAC. | | |

*Figure S1: Genotypes of patient participants*
